# Exploratory In Silico Evaluation of Microencapsulation Matrices for Indomethacin

**DOI:** 10.3390/molecules31203674

**Published:** 2026-10-14

**Authors:** Karen Isela Vargas Rubio, Efrén Delgado, Patricia Cabrales-Arellano, Claudia Ivette Gamboa Gómez, Juan Antonio Rojas-Contreras, Rubén Guerrero Rivera, Víctor Jesús Martínez Gómez, Itza Nallely Cordero Soto, María Gorety Contreras Hernández, Damián Reyes Jáquez

**Affiliations:** 1 Department of Chemical and Biochemical Engineering, National Technological Institute of Mexico (TecNM)—Durango Institute of Technology (ITD), Blvd. Felipe Pescador 1830, Nueva Vizcaya, Durango 34080, Durango, Mexico; 13040844@itdurango.edu.mx (K.I.V.R.); jrojas@itdurango.edu.mx (J.A.R.-C.); v.martinez@itdurango.edu.mx (V.J.M.G.); itza.cordero@itdurango.edu.mx (I.N.C.S.); mgorety@itdurango.edu.mx (M.G.C.H.); 2 Food Science and Technology, Department of Family and Consumer Sciences, New Mexico State University, P.O. Box 30001, Las Cruces, NM 88003, USA; 3 Department of Biology, Eastern New Mexico University, 1500 S Ave K, Portales, NM 88130, USA; cristian.cabralesarellano@enmu.edu; 4 Biomedical Research Unit, Instituto Mexicano del Seguro Social, Canoas 100, Durango 34067, Durango, Mexico; clau140382@hotmail.com; 5 Department of Electrical and Electronic Engineering, National Technological Institute of Mexico (TecNM)—Durango Institute of Technology (ITD), Blvd. Felipe Pescador 1830, Nueva Vizcaya, Durango 34080, Durango, Mexico; rubenguerrero@itdurango.edu.mx

**Keywords:** indomethacin, microencapsulation, molecular dynamics, thermodynamic compatibility, Hildebrand solubility parameter, molecular docking

## Abstract

Nonsteroidal anti-inflammatory drugs (NSAIDs), such as indomethacin (ID), may produce gastrointestinal adverse effects associated with cyclooxygenase-1 (COX-1) inhibition, motivating the investigation of microencapsulation strategies for modulating drug release. However, protein-based encapsulating materials remain comparatively underexplored using molecular simulation relative to synthetic polymers. In this study, the interaction tendencies of ID with cottonseed flour protein (CFP) and maltodextrin (MD) as potential encapsulating matrices were explored in silico using molecular dynamics simulations under NPT and NVT ensembles, cohesive energy density and Hildebrand solubility parameter (δ) calculations, the Flory–Huggins interaction parameter (χ), and mixing energy (E_mix_). The protein cohesive properties exhibited substantial force-field dependence, while the absolute χ values obtained for the binary systems were considerably higher than those reported for comparable drug–excipient systems, limiting their quantitative interpretation as predictors of miscibility. Nevertheless, within the same computational framework, CFP–ID exhibited lower χ and E_mix_ values than MD–ID, indicating comparatively less unfavorable calculated interactions. As a complementary analysis independent of the formulation assessment, molecular docking of free ID against COX-1 and COX-2 showed a relative computational trend qualitatively consistent with published enzyme-inhibition data. Overall, the results support the use of the present workflow as an exploratory computational pre-screening approach and identify CFP as the candidate warranting further investigation among the two encapsulating materials evaluated, subject to experimental validation.

## 1. Introduction

Nonsteroidal anti-inflammatory drugs (NSAIDs) are among the most widely used pharmacological agents for the management of pain and inflammation owing to their analgesic, anti-inflammatory, and antipyretic properties, with clinical applications ranging from primary dysmenorrhea to rheumatic diseases. Their use has steadily increased over recent decades as a consequence of rising life expectancy and the growing prevalence of chronic pain-related disorders [1,2,3]. NSAIDs are estimated to account for approximately 8% of medical prescriptions worldwide, while their over-the-counter use continues to increase, with nearly 26% of patients exceeding the recommended dosage [4].

The therapeutic activity of NSAIDs is primarily mediated through the inhibition of cyclooxygenase-1 and cyclooxygenase-2 (COX-1 and COX-2), enzymes involved in prostaglandin biosynthesis. Although this mechanism underlies their anti-inflammatory activity, it is also associated with a substantial proportion of their adverse effects, particularly those affecting the gastrointestinal (GI) tract. More than 40% of patients receiving conventional NSAIDs experience upper gastrointestinal symptoms during treatment, and nearly 50% may develop ulcer-related complications without previous clinical manifestations [2]. Consequently, long-term use of conventional NSAIDs can be limited by gastrointestinal toxicity. Inhibition of COX-1 reduces gastric mucus and bicarbonate secretion, weakening the natural defense mechanisms of the gastric mucosa and promoting the development of erosions, ulcers, and gastrointestinal bleeding [5]. Furthermore, studies conducted in specific patient populations have reported a three- to five-fold increase in the risk of gastrointestinal adverse events associated with NSAID therapy [6]. These limitations highlight the need for drug-delivery systems capable of modulating the release and pharmacokinetic profile of NSAIDs while maintaining their therapeutic potential.

Microencapsulation has emerged as a promising strategy for pharmaceutical delivery by protecting active compounds from degradation and enabling modulation of their release over time or at specific target sites [7,8]. In the case of NSAIDs, microcapsule-based delivery systems capable of sustained drug release under simulated biological conditions have been reported, including formulations incorporating multiple active compounds within a single delivery platform [9]. In addition, different oral formulations based on polymeric matrices have been investigated, encompassing both biodegradable synthetic polymers and naturally derived macromolecules, particularly proteins and polysaccharides, because of their biocompatibility, biodegradability, and ability to modulate drug-release kinetics [10,11]. Among these biomaterials, maltodextrin (MD) and cottonseed flour protein (CFP) have previously been investigated as microencapsulating agents [12,13], supporting their potential application as naturally derived matrices for formulation development.

Maltodextrin is widely employed as a wall material in microencapsulation owing to its high aqueous solubility, low viscosity, film-forming capacity, and ability to protect encapsulated compounds from oxidative degradation [14]. Vegetable globular proteins, including vicilin-type storage proteins, have attracted increasing interest as encapsulating matrices due to their biocompatibility, biodegradability, amphiphilic character, and capacity to form protective gels under gastrointestinal pH conditions [15]. Taken together, these complementary physicochemical characteristics support the selection of maltodextrin and CFP as candidate naturally derived matrices for oral drug-delivery applications.

In parallel with experimental advances, computational pharmaceutics has emerged as a field integrating multiscale modeling, molecular dynamics, and other in silico approaches to support rational pharmaceutical formulation design and reduce dependence on traditional trial-and-error strategies, which can be costly and time-consuming [16,17,18]. Within this framework, in silico methodologies can provide molecular-level insight into the interactions governing the stability and compatibility of pharmaceutical systems that may be difficult to characterize exclusively through experimental techniques [16,17,18].

Despite these advances, molecular dynamics investigations addressing drug–carrier miscibility through cohesive energy density calculations have predominantly focused on synthetic polymer systems [17,19], and the application of atomistic simulation workflows to naturally derived protein matrices as encapsulants for NSAIDs remains largely unexplored. This gap constitutes the primary motivation for the present study.

Among these approaches, molecular dynamics simulations (MDS) have been used to investigate drug–carrier interactions and miscibility through parameters such as cohesive energy density (CED) and the corresponding Hildebrand and Hansen solubility parameters [19,20]. These descriptors can assist in the preliminary assessment of thermodynamic compatibility among formulation components prior to experimental validation, potentially reducing development time and associated costs.

Atomistic simulations of proteins as encapsulation matrices involve additional methodological challenges, particularly regarding the assignment of force fields suitable for complex folded structures, the treatment of disulfide bridges and residual functional groups, and the construction of representative simulation cells that preserve the structural organization and density of the system. These considerations are not always addressed in standard molecular-modeling workflows, particularly those commonly applied to synthetic polymers and small molecules [20]. Accordingly, the present study aimed to explore in silico the interaction tendencies of indomethacin (ID) with a protein-based encapsulating material, cottonseed flour protein (CFP; vicilin), and a polysaccharide-based material, maltodextrin (MD), using molecular dynamics simulations and thermodynamic compatibility descriptors. Indomethacin was selected as the model NSAID owing to its well-characterized gastrointestinal toxicity profile and documented inhibitory activity toward COX-1 and COX-2, making it a relevant model compound for exploring microencapsulation strategies. A computational workflow adapted for protein macromolecules was additionally implemented to enable their incorporation into the molecular simulation framework. Molecular docking of free ID with COX-1 and COX-2 was performed as a complementary analysis, independent of the ID–encapsulant compatibility assessment.

## 2. Results

### 2.1. Molecular Structures

**Figure 1:**
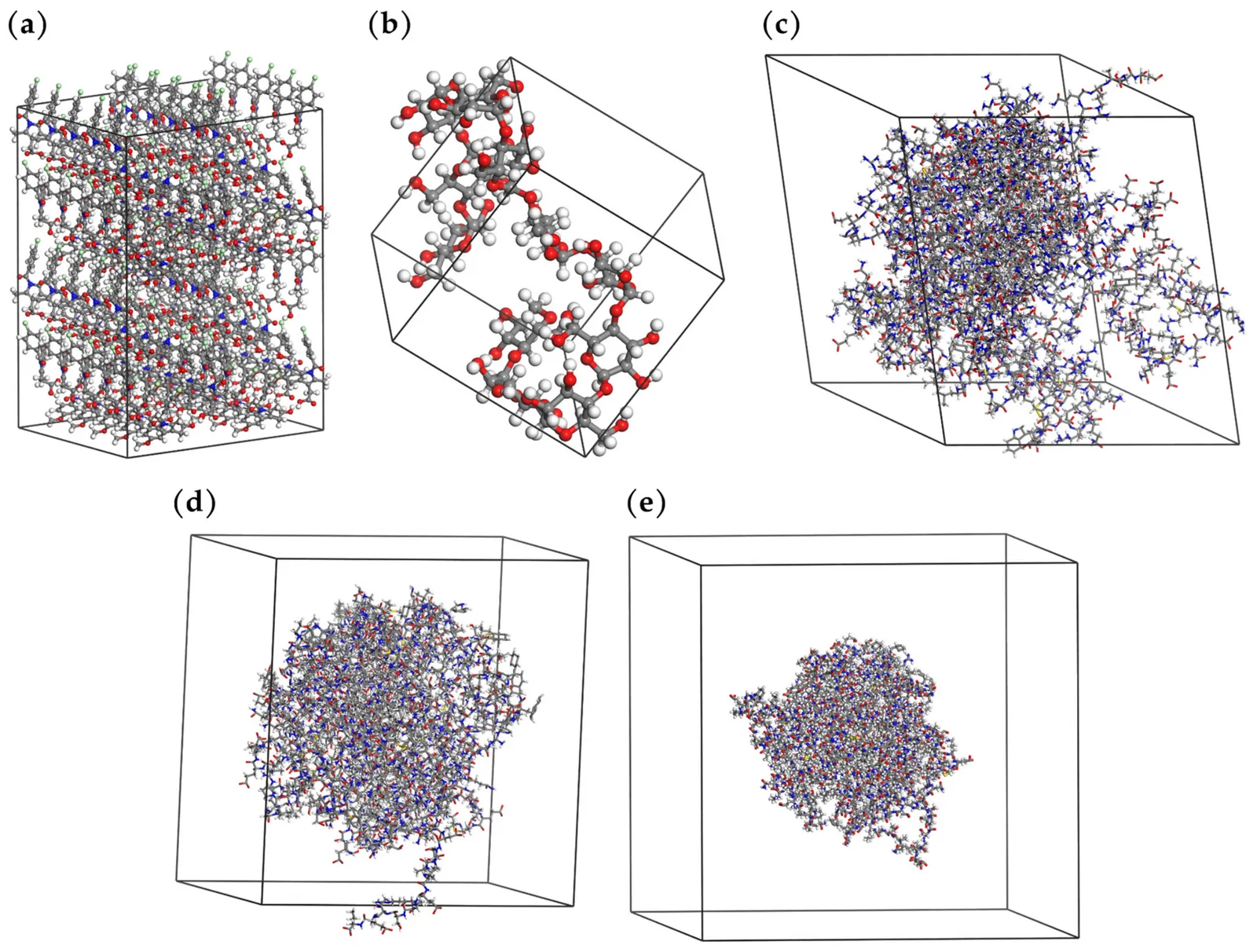
Molecular structures used in the study: (**a**) indomethacin (5 × 2 × 2 crystalline supercell containing 120 molecules of the α polymorph); (**b**) maltodextrin; (**c**) cottonseed flour protein; (**d**) cyclooxygenase-1; and (**e**) cyclooxygenase-2.

Based on the construction and selection procedures described in Section 4.1, the molecular structures of indomethacin (ID), maltodextrin (MD), cottonseed flour protein (CFP), cyclooxygenase-1 (COX-1), and cyclooxygenase-2 (COX-2) were obtained and prepared for the subsequent computational analyses (Figure 1). For ID, the structure corresponded to a crystalline supercell of the α polymorph (P2_1_ space group, 5 × 2 × 2, 120 molecules), reconstructed from experimental X-ray diffraction data as described in Section 4.1.1. For the protein structures, hydrogen atoms were added and the corresponding structural preparation procedures were performed prior to geometry optimization.

Once the initial structures had been prepared, geometry optimization was performed to minimize their potential energy and obtain stable molecular configurations for the subsequent simulations.

### 2.2. Geometry Optimization

Geometry optimization procedures were adapted to the characteristics of each molecular system. For the isolated ID molecule and MD, optimization was performed using the COMPASS II force field and the Smart algorithm (Table 1). In both cases, geometry optimization resulted in a substantial decrease in total energy, from 198.189 to 1.979 kcal/mol for ID and from 180,342.719 to 332.815 kcal/mol for MD. The larger energy decrease observed for MD was accompanied by a conformational rearrangement of the polysaccharide chain.

The crystalline structure of ID was additionally optimized at two scales—the unit cell and the 5 × 2 × 2 supercell. In both cases, the atomic positions were first relaxed while maintaining fixed cell parameters, followed by optimization of the cell parameters (Table 2). Changes in the cell parameters remained below 2% for the unit cell and 1% for the supercell relative to the experimental crystallographic values, indicating that the crystalline configuration was largely preserved during optimization.

For the protein structures, the DREIDING and COMPASS II force fields were evaluated in parallel (Table 3). Substantial differences were observed in the initial and final energies calculated with the two force fields. The initial COMPASS II energies were approximately 53.8–57.8 times higher than those obtained using DREIDING, with these differences predominantly associated with the van der Waals energy contribution. Because DREIDING and COMPASS II employ different parameterization schemes and energetic descriptions, the absolute energy values obtained with the two force fields were not considered directly comparable measures of protein stability or force-field performance. In addition, missing parameters were identified for the atom pair (c3l, o1^−^) when COMPASS II was applied and were automatically assigned by the software, whereas no missing parameters were reported with DREIDING.

**Figure 2:**
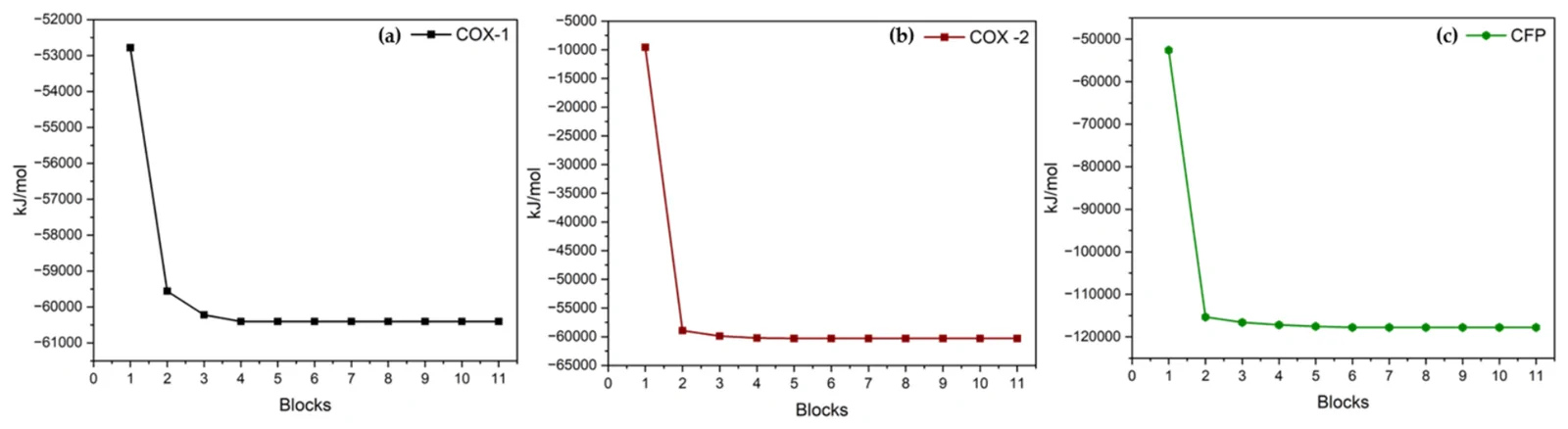
Energy profiles obtained during block-wise optimization of the protein structures: (**a**) cyclooxygenase-1; (**b**) cyclooxygenase-2; and (**c**) cottonseed flour protein.

The protein structures were initially subjected to the block-wise optimization procedure described in Section 4.1 using CHARMM-GUI and Google Colab. The corresponding energy profiles are shown in Figure 2.

### 2.3. Molecular Dynamics

#### 2.3.1. NVT Ensemble

The thermodynamic properties obtained under the NVT ensemble are summarized in Table 4. The average temperature remained close to the target value of 303.15 K for all systems, with standard deviations ranging from 14.3 to 22.0 K. Substantial differences in potential and total energy were observed among the protein systems depending on the force field employed. Because DREIDING and COMPASS II differ in their parameterization and energetic descriptions, their absolute energy values were not interpreted as indicators of relative protein stability or force-field performance. For the ID crystalline supercell, the density remained fixed at 1.458 g/cm^3^, while the average potential and total energies were 44.329 and 81.370 kcal/mol per molecule, respectively.

#### 2.3.2. NPT Ensemble

The thermodynamic properties obtained under the NPT ensemble are summarized in Table 5. The average temperature remained close to the target value of 303.15 K for all systems, with standard deviations ranging from 14.4 to 24.0 K. As observed under the NVT ensemble, substantial differences in potential energy were observed for the protein structures depending on the force field employed. Because DREIDING and COMPASS II differ in their parameterization and energetic descriptions, these absolute energy values were not interpreted as indicators of relative protein stability or force-field performance. For the ID crystalline supercell, the average potential and total energies were 45.021 and 82.057 kcal/mol per molecule, respectively. During the NPT simulation, the volume of the ID supercell increased from 48,887.5 to 51,763.2 Å^3^, resulting in an average density of 1.380 g/cm^3^.

#### 2.3.3. Cohesive Energy Density (CED)

**Figure 3:**
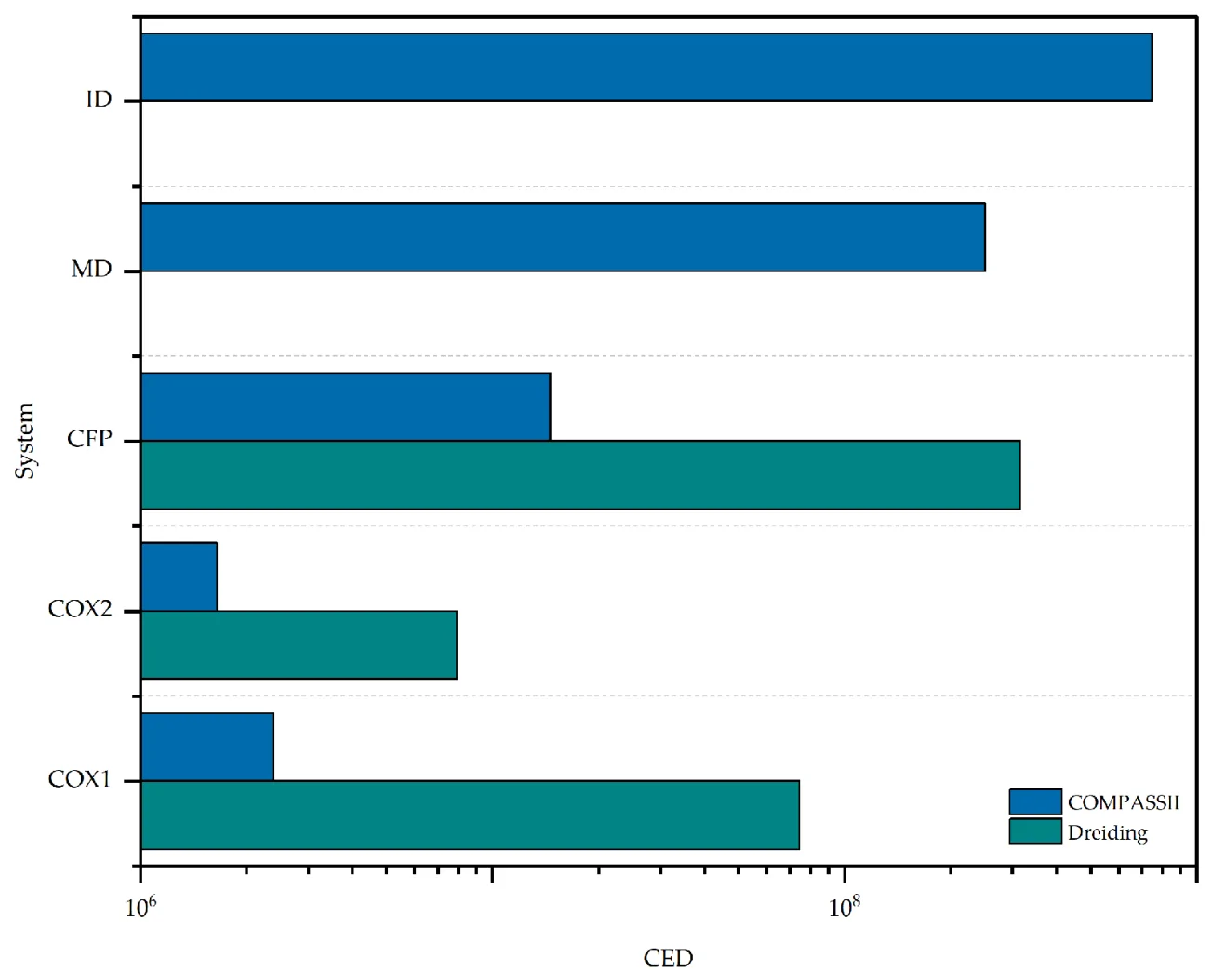
Comparison of the cohesive energy density of the systems studied.

The cohesive energy density (CED) was calculated from the NVT trajectory for each evaluated system. The resulting values are presented in Table 6 and compared graphically in Figure 3.

ID exhibited the highest CED among the systems evaluated using COMPASS II (7.409 × 10^8^ J/m^3^), followed by MD (2.510 × 10^8^ J/m^3^). For ID, the van der Waals contribution accounted for approximately 72% of the CED, while the electrostatic and residual contributions were 181.5 × 10^6^ and 28.30 × 10^6^ J/m^3^, respectively. For the protein systems, the calculated CED values showed substantial dependence on the force field employed. CFP, for example, exhibited CED values of 3.162 × 10^8^ and 1.458 × 10^7^ J/m^3^ using DREIDING and COMPASS II, respectively. CED values obtained with COMPASS II were also lower than those obtained with DREIDING for COX-1 and COX-2. These differences demonstrate the sensitivity of the calculated protein cohesive properties to force-field parameterization.

### 2.4. Thermodynamic Compatibility

The thermodynamic interaction tendencies between indomethacin (ID) and the encapsulating agents were explored using three complementary descriptors: the Hildebrand solubility parameter (δ), the Flory–Huggins interaction parameter (χ), and the mixing energy (E_mix_).

#### 2.4.1. Hildebrand Solubility Parameter (δ)

The Hildebrand solubility parameter (δ) values calculated for the evaluated structures are presented in Table 7. These values were derived from the cohesive energy density obtained from the NVT trajectories.

ID exhibited a δ value of 27.219 (J/cm^3^)^0.5^, while MD exhibited a value of 15.814 (J/cm^3^)^0.5^ using COMPASS II. For the protein systems, the calculated δ values showed a marked dependence on the force field employed. In particular, CFP exhibited δ values of 17.783 and 3.819 (J/cm^3^)^0.5^ using DREIDING and COMPASS II, respectively. Similar force-field-dependent differences were observed for COX-1 and COX-2. Given the magnitude of these differences, the absolute δ values obtained for the protein systems were not considered directly interchangeable quantitative estimates of thermodynamic compatibility. Instead, the complete set of values was retained to characterize the sensitivity of the calculated cohesive properties to force-field parameterization.

#### 2.4.2. Flory–Huggins Interaction Parameter (χ) and Mixing Energy (E_mix_)

The E_mix_ and χ values calculated for the ID–encapsulant binary systems using the Blends module of Materials Studio are presented in Table 8.

Both binary systems exhibited positive E_mix_ and χ values. The MD–ID system exhibited higher values of both χ (41.239) and E_mix_ (24.421 kcal/mol) than the CFP–ID system (χ = 35.115; E_mix_ = 20.795 kcal/mol). Given the magnitude of the absolute χ values, these results were not interpreted as quantitative predictions of miscibility. However, because both systems were evaluated using the same Blends computational framework, the relative ordering of the calculated descriptors indicates comparatively less unfavorable interactions for CFP–ID than for MD–ID.

### 2.5. Molecular Docking

**Figure 4:**
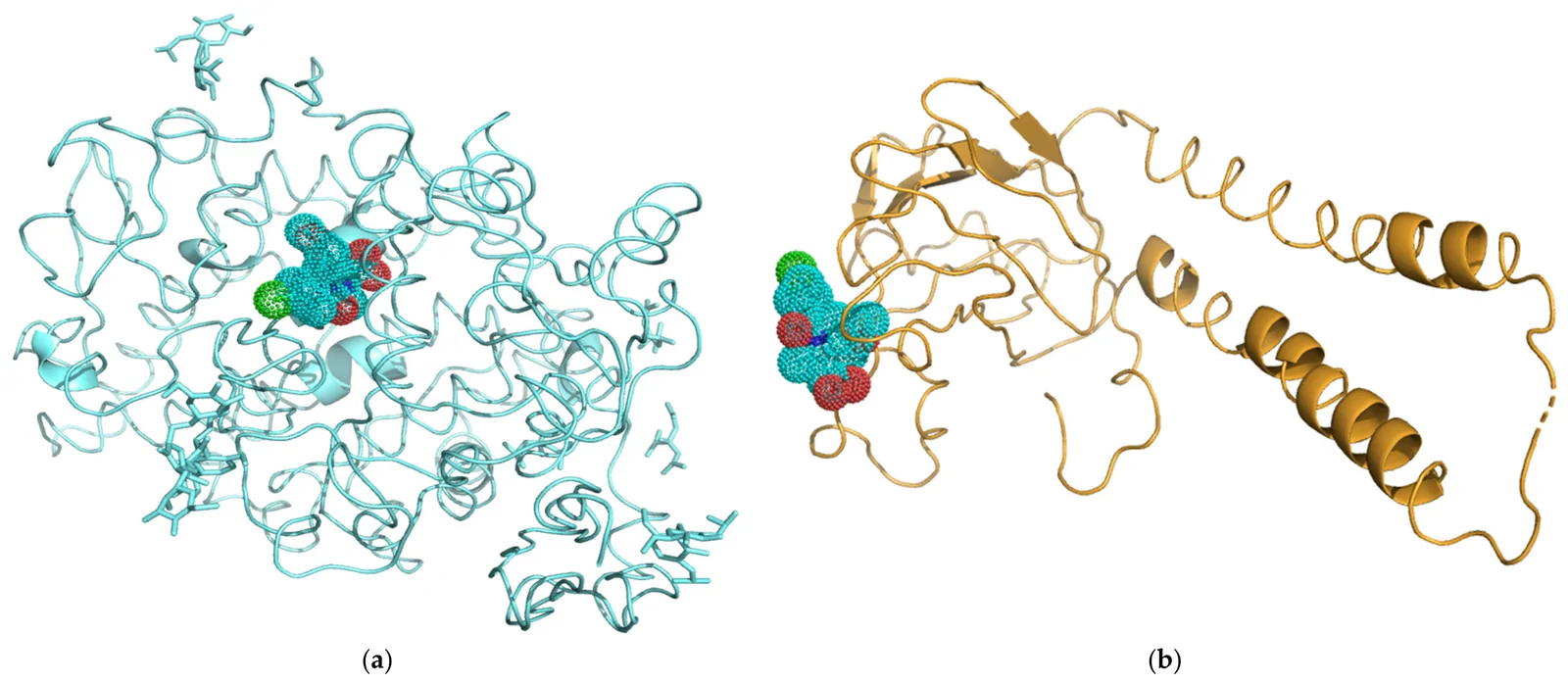
Top-ranked molecular docking poses obtained using HDOCK for (**a**) the COX-1–ID complex and (**b**) the COX-2–ID complex.

Molecular docking of ID with COX-1 and COX-2 generated 100 poses for each system, from which the 10 top-ranked poses were analyzed (Table 9). For the COX-1–ID complex, docking scores ranged from −152.45 to −122.64, with confidence scores ranging from 0.5235 to 0.7317. The top-ranked pose exhibited a docking score of −152.45 and a confidence score of 0.7317 (Figure 4a). For the COX-2–ID complex, docking scores ranged from −121.24 to −93.89, with confidence scores ranging from 0.3508 to 0.5142. The top-ranked pose exhibited a docking score of −121.24 and a confidence score of 0.5142 (Figure 4b).

The difference between the top-ranked poses of the two systems was 31.21 docking-score units and 0.2175 in confidence score. Among the two complexes, only the top-ranked COX-1–ID pose exceeded the HDOCK confidence-score threshold of 0.7. These scores were used for comparative purposes within the HDOCK analysis and were not interpreted as quantitative estimates of binding affinity or pharmacological efficacy.

## 3. Discussion

### 3.1. Geometry Optimization

The differences in energy observed between DREIDING and COMPASS II for the protein structures should be considered in the context of the different parameterization strategies of these force fields. COMPASS II was developed for condensed-phase applications, with van der Waals parameters fitted using experimental equilibrium densities and cohesive energies [21], whereas DREIDING is a generic force field based primarily on atomic hybridization and connectivity [22]. A comparative study evaluating 324 force-field protocols across 235 organic crystals within the same computational environment used in the present work identified COMPASS II as the best-performing force field overall and particularly recommended it for pharmaceutical crystals [23]. However, this evidence concerns predominantly organic crystalline systems and does not establish COMPASS II as an optimal force field for protein simulations. Accordingly, the substantial differences observed between DREIDING and COMPASS II for the protein systems are interpreted here as evidence of force-field sensitivity rather than as evidence of superior performance by either force field. The results obtained with both force fields were therefore retained to characterize this methodological sensitivity.

For ID, the crystalline cell was reconstructed using the experimental lattice parameters and atomic coordinates of the α polymorph [24]. Following geometry optimization with COMPASS II, changes in the cell parameters remained below 2% relative to the experimental values, indicating limited distortion of the initial crystalline structure and preservation of the principal packing characteristics of this polymorph. These results support the use of the optimized crystalline structure as the starting configuration for the subsequent MDS.

### 3.2. NPT and NVT Ensembles

The maintenance of the average temperature near the target value of 303.15 K across all systems indicates adequate thermal control during the simulations, regardless of molecular size or structural complexity. Differences in the sign and magnitude of the potential energies were observed among chemically different systems and between the DREIDING and COMPASS II calculations for the protein structures. Because these force fields differ in their functional forms and parameterization, their absolute potential-energy values should not be directly compared as indicators of relative protein stability or force-field performance. Accordingly, the observed energetic differences are interpreted primarily as evidence of the sensitivity of the protein calculations to force-field selection.

For crystalline ID, the average density obtained under the NPT ensemble (1.380 ± 0.014 g/cm^3^) was close to that calculated from the experimental crystallographic data for the α polymorph (Z = 6, V = 2501.879 Å^3^ [24]). The corresponding crystallographic density was 1.425 g/cm^3^, representing a difference of approximately 3.2% from the simulated value. This agreement supports the ability of the reconstructed crystalline system to reasonably reproduce this experimental solid-state property. Furthermore, the normalized potential and total energy values obtained for ID under the NPT and NVT ensembles differed by less than 1.6%, providing additional evidence of consistency between the two simulation conditions.

### 3.3. Cohesive Energy Density

ID exhibited the highest CED among the systems evaluated using COMPASS II (7.409 × 10^8^ J/m^3^), with van der Waals interactions accounting for approximately 72% of the total cohesive contribution. This predominance is consistent with the aromatic and chlorinated molecular structure of ID, which provides a substantial molecular surface for dispersion interactions. The CED obtained for the experimentally supported crystalline ID structure provides the basis for calculation of its Hildebrand solubility parameter. In contrast, the protein systems exhibited substantial force-field dependence in their calculated CED values. For example, CFP exhibited CED values of 3.162 × 10^8^ and 1.458 × 10^7^ J/m^3^ using DREIDING and COMPASS II, respectively. Because the Hildebrand solubility parameter is derived directly from CED, this force-field sensitivity propagates to the calculated δ values and limits their quantitative interpretation for the protein systems.

An additional limitation affecting the protein CED values arises from the construction of the protein simulation cells. Each protein was centered within a 150 × 150 × 150 Å periodic cell, resulting in substantial unoccupied volume around the isolated macromolecule. Consequently, when the full simulation-cell volume is used in the CED calculation, the resulting CED and derived δ values are inherently dependent on the selected cell dimensions and should not be interpreted as intrinsic material properties of the proteins. This cell-volume dependence, together with the pronounced force-field sensitivity described above, further limits the quantitative interpretation of the protein CED and δ values. Accordingly, these descriptors are retained primarily to characterize methodological sensitivity within the present exploratory computational workflow.

### 3.4. Hildebrand Solubility Parameter

The δ value calculated for ID (27.219 (J/cm^3^)^0.5^) was of the same order of magnitude as the experimental estimate reported using the Extended Hildebrand Solubility Approach (EHSA). In that study, maximum indomethacin solubility in 1,4-dioxane–water mixtures occurred at a solvent solubility parameter of 21.9 MPa^0.5^, which was used as an experimental approximation of the effective solubility parameter of the drug [25]. The approximately 24% difference between the experimental estimate and the value obtained in the present study is reasonable considering the methodological differences between the two approaches. The experimental value was derived from liquid-phase solubility data at 298.15 K, with a reported methodological uncertainty of up to 30%, whereas the present computational value was obtained for crystalline ID at 303.15 K. Differences in molecular organization and intermolecular interactions between the crystalline and solution states may therefore contribute to the observed discrepancy.

In contrast to the comparatively close agreement observed for ID, the protein systems exhibited a pronounced dependence of the calculated δ values on the force field employed. For example, CFP exhibited δ values of 17.783 and 3.819 (J/cm^3^)^0.5^ using DREIDING and COMPASS II, respectively, with substantial differences also observed for COX-1 and COX-2. Because δ is derived directly from CED, these differences reflect the force-field sensitivity observed for the cohesive properties of the protein systems. Consequently, the absolute protein δ values obtained with DREIDING and COMPASS II should not be considered directly interchangeable quantitative estimates or used independently to establish drug–protein miscibility. Instead, their comparison is retained to explicitly demonstrate the sensitivity of these calculated descriptors to force-field parameterization within the present computational workflow.

### 3.5. Flory–Huggins Interaction Parameter and Mixing Energy

The χ values obtained for CFP–ID (35.115) and MD–ID (41.239) were considerably higher than values reported for comparable drug–excipient systems evaluated using the same Materials Studio module, for which χ values typically range from approximately −6 to +6 [26]. This substantial difference indicates that the absolute χ values obtained in the present study should not be interpreted as quantitative predictions of miscibility. Instead, they are considered here only as comparative descriptors of interaction tendency within the same Blends computational framework.

Several characteristics of the computational model may have contributed to the magnitude of these values. In particular, the MD oligomer was not configured using the “terminal atoms without contact” option in the Blends module, which is intended to represent longer polymer chains using shorter molecular fragments. In addition, the substantial difference in molecular size between the Base components (CFP and MD) and the Screen component (ID) may affect the applicability of the Flory–Huggins approximation to these systems.

Taken together, these limitations preclude interpretation of the absolute χ values as quantitative predictions of miscibility. Nevertheless, because CFP–ID and MD–ID were evaluated using the same Blends workflow and simulation conditions, the calculations can provide a comparative indication of interaction tendency within the present model. Both χ and E_mix_ showed the same relative ordering, with lower values for CFP–ID than for MD–ID. Thus, the principal information derived from these calculations is not that either system is predicted to be miscible, but rather that CFP–ID exhibited comparatively less unfavorable calculated interactions than MD–ID. This relative trend should be regarded as an exploratory pre-screening result requiring validation using experimentally prepared formulations.

### 3.6. Molecular Docking

The docking analysis provided a complementary molecular characterization of the interaction of free ID with its established pharmacological targets, independent of the thermodynamic evaluation of the encapsulating matrices. The top-ranked ID–COX-1 pose exhibited a more negative docking score and higher confidence score than the corresponding ID–COX-2 pose. This relative computational trend is qualitatively consistent with experimental enzyme-inhibition data, as indomethacin has been reported to exhibit an IC_50_ of 27 nM against ovine COX-1, compared with 127 and 180 nM against murine and human COX-2, respectively, corresponding to approximately 5- to 7-fold greater inhibitory potency toward COX-1 despite its clinical classification as a nonselective NSAID [27]. This qualitative agreement should not be interpreted as validation of the docking scores as quantitative measures of binding affinity or pharmacological efficacy.

Structural differences between the two COX isoforms provide additional context for the observed computational trend. The COX-2 active site has been reported to be approximately 25% larger than that of COX-1 [27]. However, because the present analysis employed global docking without binding-site restraints, the differences in docking and confidence scores should not be attributed to a specific active-site interaction mechanism. The structural differences between the isoforms are therefore considered contextual information rather than a mechanistic explanation for the docking results.

Importantly, this docking analysis addresses the interaction of free ID with its pharmacological targets and is independent of the thermodynamic evaluation of ID–encapsulant compatibility. It therefore does not provide evidence of drug–matrix miscibility, formulation performance, or preservation of pharmacological activity following microencapsulation. Rather, it is retained as a complementary molecular characterization of ID, with the docking results interpreted qualitatively and within the limitations of the single-platform computational approach.

## 4. Materials and Methods

### 4.1. Molecular Structure

The molecular structures used in this study were selected according to their roles in the evaluated systems. Indomethacin (ID) was considered the active pharmaceutical ingredient, maltodextrin (MD) and cottonseed flour protein (CFP; Vicilin C72) were evaluated as encapsulating agents, and COX-1 and COX-2 were included as pharmacological targets.

#### 4.1.1. Indomethacin

The three-dimensional structure of indomethacin (1-(4-chlorobenzoyl)-5-methoxy-2-methyl-1H-indole-3-acetic acid) was constructed using the Visualizer module of Materials Studio 2020 (version 20.1.0.2728; Dassault Systèmes BIOVIA, San Diego, CA, USA) and subsequently optimized using the Forcite module with the COMPASS II force field. In addition, the crystalline form of indomethacin was identified by powder X-ray diffraction (PXRD) using the contents of commercial indomethacin capsules (25 mg; Laboratorios Silanes, S.A. de C.V., Toluca, Mexico; lot 26B795V2). PXRD patterns were recorded on a Rigaku MiniFlex diffractometer (Rigaku Corporation, Tokyo, Japan) equipped with a SC-70 scintillation counter detector, operating at 40 kV and 15 mA. The diffraction pattern was identified as corresponding to the α polymorph. This polymorph belongs to the monoclinic crystal system and the P2_1_ space group, with unit-cell parameters *a* = 5.4616 Å, *b* = 25.310 Å, *c* = 18.152 Å, β = 94.380°, and Z = 6 [26]. Based on this experimental identification, the corresponding crystallographic information file (CIF) was obtained from the Crystallography Open Database (COD 4115393, accessed on 23 September 2026) [24]. The CIF was imported into Materials Studio to reconstruct the crystal unit cell using the experimental atomic coordinates and was subsequently subjected to the geometry-optimization procedure described in Section 4.2. ID is a nonselective NSAID that inhibits both COX-1 and COX-2, thereby interfering with prostaglandin and thromboxane synthesis from arachidonic acid [28,29,30].

#### 4.1.2. Maltodextrin

Maltodextrin was selected as an encapsulating agent because of its widespread use as a wall material in food and pharmaceutical microencapsulation systems. Its favorable properties include high water solubility, low viscosity at high solids concentrations, relatively low cost, thermal and acid stability, freeze resistance, and the ability to protect encapsulated compounds against oxidative processes [14]. Its molecular structure was constructed in Materials Studio 2020 using the Visualizer module and the Homopolymer tool. A representative oligomeric chain composed of glucose units linked by α-1,4 glycosidic bonds was generated and subsequently subjected to geometry optimization.

#### 4.1.3. Cottonseed Flour Protein (CFP/Vicilin C72)

The three-dimensional structure of cottonseed vicilin C72 (*Gossypium hirsutum*; UniProt ID: P09801) was obtained from the AlphaFold Protein Structure Database (https://alphafold.ebi.ac.uk; accessed on 27 June 2025) [31,32], corresponding to entry AF-P09801-F1. This model was selected because it contains the complete 588-amino-acid sequence without missing residues or non-protein heteroatoms, thereby facilitating its preparation for MDS. Vicilin C72 belongs to the 7S globulin family and constitutes one of the major protein fractions of cottonseed [33].

#### 4.1.4. Cyclooxygenase-1

The three-dimensional structure of human cyclooxygenase-1 (prostaglandin G/H synthase-1; PTGS1; UniProt ID: P23219) was obtained from the AlphaFold Protein Structure Database, corresponding to entry AF-P23219-F1 [34]. The predicted structure contains 599 amino acids and exhibits high prediction confidence (pLDDT > 90) throughout the central globular region. The AlphaFold-derived model was selected because alternative available structures contained missing residues and non-protein heteroatoms that complicated their preparation for MDS. COX-1 is a major pharmacological target of indomethacin and other nonselective NSAIDs [35].

#### 4.1.5. Cyclooxygenase-2

The three-dimensional structure of human cyclooxygenase-2 (prostaglandin G/H synthase-2; PTGS2; UniProt ID: P35354) was obtained from the AlphaFold Protein Structure Database, corresponding to entry AF-P35354-F1 [36]. The structure contains 604 amino acids and, similarly to COX-1, belongs to the prostaglandin G/H synthase family. The two isoforms differ in their expression patterns and in structural features of their active sites that contribute to differences in pharmacological selectivity [35]. Both enzymes were included because indomethacin inhibits COX-1 and COX-2 as a nonselective NSAID.

### 4.2. Geometry Optimization

Geometry optimization was performed for all molecular structures to obtain energetically minimized configurations suitable as starting structures for subsequent computational analyses and MDS. The geometry optimizations performed in Materials Studio/Forcite were carried out in vacuo, without explicit or implicit solvent. For the protein systems, this step followed the implicit-solvent energy-minimization procedure described in Section 4.2.2. Different optimization strategies were employed according to the size, molecular nature, and structural complexity of each system.

#### 4.2.1. Indomethacin and Maltodextrin

The ID and MD structures were geometrically optimized in Materials Studio 2020 using the Forcite module and the COMPASS II force field [21]. The Smart algorithm was employed with an energy convergence criterion of 0.001 kcal/mol and a maximum of 5000 iterations. Electrostatic interactions were calculated using the Ewald summation method, whereas van der Waals interactions were treated using an atom-based summation scheme with a cutoff distance of 12.5 Å. Atomic charges were assigned using the force-field charge assignment scheme implemented in COMPASS II.

For crystalline ID, the structure reconstructed from the CIF file described in Section 4.1.1 was initially optimized while maintaining fixed unit-cell parameters. A second optimization stage was subsequently performed in which relaxation of the cell parameters was allowed. The optimized unit cell was then replicated to construct a 5 × 2 × 2 supercell, corresponding to 20 unit cells. Because the ID unit cell contains six molecules (Z = 6), the resulting supercell contained 120 ID molecules. The final supercell dimensions were approximately 27.74 × 50.02 × 35.29 Å. These dimensions allowed the use of a 12.5 Å cutoff for nonbonded interactions while maintaining each cell dimension above twice the cutoff distance. The optimized supercell was used as the starting configuration for subsequent MDS and CED calculations.

The MD structure was optimized using the same computational protocol, including the COMPASS II force field, Smart algorithm, convergence criterion, and nonbonded interaction settings.

#### 4.2.2. Proteins (COX-1, COX-2 and CFP)

Owing to the size and structural complexity of the protein systems, the structures could not be directly incorporated into simulation cells using the Amorphous Cell module of Materials Studio. Therefore, a multistep computational protocol was implemented for structural preparation, energy minimization, cell construction, and final geometry optimization.

Stage 1. Structural preparation using CHARMM-GUI

The AlphaFold-derived structures of COX-1, COX-2, and CFP were processed using the PDB Reader & Manipulator tools of CHARMM-GUI (version 3.7; https://www.charmm-gui.org; accessed between 24 June and 14 August 2026) [37,38,39,40,41,42]. Hydrogen atoms were added using standard geometries, N-terminal (NTER) and C-terminal (CTER) groups were assigned, and disulfide bonds identified from the primary sequences were defined. CHARMM topology files were subsequently generated using the CHARMM36m force field [43]. Assignment of atomic types and structural parameters was verified by performing a single-point energy calculation in CHARMM (version c47a2).

Stage 2. Energy minimization using OpenMM/Google Colaboratory

Input files for energy minimization were generated using the Implicit Solvent Modeler module of CHARMM-GUI with the GBMV (Generalized Born with Molecular Volume) model, a salt concentration of 0.0 M, and a temperature of 303.15 K. Energy minimization was subsequently performed using OpenMM 8.5.2 [44] in a Python 3.12 environment implemented in Google Colaboratory (Google LLC, Mountain View, CA, USA) employing the HCT (Hawkins–Cramer–Truhlar) implicit-solvent model without bond constraints and with an ionic concentration of 0.0 M. Minimization was conducted in 10 blocks of 50 iterations, and the structure was saved after each block to monitor the evolution of potential energy. Convergence was assessed based on stabilization of the potential energy between consecutive blocks.

Stage 3. Cell Construction

The minimized protein structures were centered using the OpenMM Python library such that each protein was positioned at the geometric center of the simulation cell. A cubic cell without crystallographic symmetry and with dimensions of 150 × 150 × 150 Å was subsequently constructed in Materials Studio using the Build Crystal module [45,46]. Periodic boundary conditions were applied in all three dimensions. The centered protein was inserted into the cell, and the system was reconstructed using the Rebuild Crystal tool.

Stage 4. Geometry Optimization in Materials Studio

Protein structures within the simulation cells were geometrically optimized using the Forcite module of Materials Studio with the DREIDING [22] and COMPASS II [21] force fields evaluated in parallel. These force fields were employed at this stage because the subsequent NVT/NPT and CED calculations were implemented within the Materials Studio/Forcite workflow. Their parallel evaluation was intended to assess the sensitivity of the calculated protein descriptors to force-field parameterization within this computational framework, rather than to establish either force field as an optimal biomolecular force field. Gasteiger charges were used with both force fields to permit comparison under a common charge-assignment scheme. The Smart algorithm was employed with a maximum of 5000 iterations, an energy convergence criterion of 0.001 kcal/mol, and a force convergence criterion of 0.5 kcal/mol/Å. Electrostatic interactions were treated using the Ewald summation method, whereas van der Waals interactions were calculated using an atom-based truncation scheme with a cutoff distance of 12.5 Å.

DREIDING provides general parameters covering a broad range of atom types [22]. During application of COMPASS II, missing bond, angle, and torsion parameters were identified for specific atom types and were automatically assigned by the software. Results obtained using both force fields were retained to explicitly evaluate force-field sensitivity in the system energies, NVT and NPT simulations, and CED calculations. Because DREIDING and COMPASS II employ different parameterization schemes and energetic descriptions, their absolute energy values were not considered directly comparable measures of protein stability or force-field performance.

### 4.3. Molecular Dynamics

Molecular dynamics simulations (MDS) were performed using the NVT and NPT ensembles at a target temperature of 303.15 K, with a time step of 1.0 fs and a total simulation time of 100 ps (100,000 steps) for each ensemble. The NVT stage was first conducted at constant volume using the Nosé thermostat [47,48], with trajectory frames saved every 5000 steps, to thermally equilibrate the systems. Subsequently, NPT simulations were performed at a pressure of 0.0001 GPa (approximately 1 atm) using the Nosé thermostat and the Berendsen barostat, with a pressure decay constant of 0.1 ps, allowing the volume and density of each system to adjust under the specified temperature and pressure conditions.

Equilibration was assessed from the stabilization of temperature and potential energy during the second half of the NVT trajectory and from the stabilization of density during the NPT trajectory [49]. Potential-energy convergence profiles for the protein systems evaluated with DREIDING and COMPASS II are provided in Supplementary Material File S1 (Figures S1–S6). These profiles showed an initial energetic relaxation followed by a comparatively stable energy region over the remainder of the 100 ps NVT simulations. Structural RMSD profiles could not be obtained because the trajectory files required for this analysis were not retained; therefore, structural convergence could not be independently assessed by RMSD. For each system, total, potential, kinetic, and nonbonded energies were analyzed, including van der Waals, electrostatic, and hydrogen-bond contributions. Average energy values obtained from the NVT trajectories were subsequently used to calculate the cohesive energy density (CED) and Hildebrand solubility parameter (δ) according to the methodology described by Belmares et al. [50]. Given the 100 ps duration of each ensemble, these trajectories were used for exploratory comparative analysis within the present computational workflow rather than for exhaustive conformational sampling of the protein systems.

### 4.4. Thermodynamic Interaction Analysis

The thermodynamic interaction tendencies between ID and the encapsulating agents were explored using three complementary descriptors: the Hildebrand solubility parameter (δ), the Flory–Huggins interaction parameter (χ), and the mixing energy (E_mix_). Together, these descriptors were used to comparatively assess ID–encapsulant interactions within the selected computational framework [50,51].

#### 4.4.1. Hildebrand Solubility Parameter (δ)

The Hildebrand solubility parameter (δ) was calculated from the cohesive energy density (CED) obtained using the Cohesive Energy Density analysis in the Forcite module of Materials Studio, according to Equation (1).

(1) $$\delta =\sqrt{CED}=\sqrt{\frac{E_{coh}}{V}}=\sqrt{\frac{-E_{inter}}{V}}$$

In Equation (1), the corresponding energy term represents the intermolecular cohesive energy and V represents the volume of the simulation cell. Calculations were performed using the NVT trajectory at 303.15 K, and δ values were expressed in (J/cm^3^)^0.5^. Differences between the solubility parameters of the components were considered as a conventional indicator of thermodynamic compatibility, with Δδ < 2.0 (J/cm^3^)^0.5^ generally associated with a higher likelihood of miscibility [50,52]. However, because the protein δ values obtained in the present study exhibited substantial force-field dependence, this criterion was not treated as a quantitative predictor of miscibility for the protein-containing systems.

For the protein systems, each simulation cell contained a single isolated protein molecule within the 150 × 150 × 150 Å periodic cell described in Section 4.2.2. No explicit or implicit solvent was included during the Materials Studio CED calculations; the implicit-solvent treatment described above was restricted to the initial protein-preparation stage. The volume used in Equation (1) corresponded to the full simulation-cell volume. Consequently, the protein CED and derived δ values were considered methodological descriptors within the present computational workflow rather than intrinsic condensed-phase material properties.

#### 4.4.2. Flory–Huggins Interaction Parameter (χ) and Mixing Energy (E_mix_)

The Flory–Huggins interaction parameter (χ) and mixing energy (E_mix_) were obtained using the Blends module of Materials Studio. The relationship between these parameters was evaluated according to Equation (2).

(2) $$\chi =\frac{E_{mix}}{RT}$$

In Equation (2), E_mix_ represents the mixing energy associated with the energetic difference between the mixed state and the individual components, R is the universal gas constant (8.314 J mol^−1^ K^−1^), and T is the absolute temperature (303.15 K). Lower χ values are generally associated with more favorable interactions and greater thermodynamic compatibility, whereas higher values indicate less favorable interactions and a greater tendency toward immiscibility [51,53].

The corresponding E_mix_ and χ values for the binary ID–encapsulant systems were obtained from the Blends module. Negative E_mix_ values are generally associated with energetically favorable mixing, whereas positive values indicate less favorable interactions between the components. Given the magnitude of the χ values obtained in the present study, these descriptors were used primarily for comparative assessment of CFP–ID and MD–ID within the same computational framework rather than as quantitative predictors of miscibility. The combined evaluation of δ, χ, and E_mix_ was therefore used to characterize relative ID–encapsulant interaction tendencies [50].

### 4.5. Molecular Docking

Molecular docking of indomethacin with COX-1 and COX-2 was performed as a complementary analysis to characterize the predicted interaction of free ID with these pharmacological targets and to compare its relative computational behavior toward the two cyclooxygenase isoforms. This analysis was conducted independently of the thermodynamic evaluation of ID–encapsulant interactions. Docking simulations were performed using the HDOCK online server (http://hdock.phys.hust.edu.cn; Huazhong University of Science and Technology, Wuhan, China; accessed on 25 August 2025) [54,55], which implements a hybrid algorithm combining template-based and template-free docking strategies and uses ITScorePP and ITScorePL scoring functions for protein–protein and protein–ligand interactions, respectively [55].

For the ID–COX-1 and ID–COX-2 systems, the geometrically optimized protein structures obtained from Materials Studio 2020 were used as receptors, whereas the three-dimensional structure of ID optimized using COMPASS II was used as the ligand. The structures were uploaded in PDB format, and global docking was performed without binding-site restraints, allowing the algorithm to identify potential interaction regions across the protein surface. Because this unrestricted approach explores the full protein surface rather than a predefined binding site, the resulting pose ensemble may include interactions at partially solvent-exposed surface regions. Such poses reflect the global nature of the docking search and should not, by themselves, be interpreted as evidence of biologically relevant binding.

A total of 100 docking poses were generated for each system. The 10 top-ranked poses were retained for comparative score analysis, and the top-ranked pose from each system was selected for structural visualization using Open-Source PyMOL 3.1 (Schrödinger, LLC, New York, NY, USA).

The resulting complexes were evaluated using the Docking Score and Confidence Score reported by HDOCK. The Docking Score corresponds to the energy-based score derived from the ITScore function, with more negative values indicating more favorable predicted interactions [55]. The Confidence Score is a normalized estimate of model reliability ranging from 0 to 1, with values above 0.7 considered indicative of high confidence [55]. The docking scores were used for comparative interpretation within the HDOCK framework and were not treated as quantitative estimates of binding affinity or pharmacological efficacy. The computational results were subsequently compared qualitatively with experimental data describing the relative inhibitory activity of indomethacin toward COX-1 and COX-2 reported in the literature [27,35,56].

## 5. Conclusions

The present study developed and applied a computational workflow to explore the interaction tendencies of indomethacin (ID) with cottonseed flour protein (CFP/Vicilin) and maltodextrin (MD) as potential encapsulating matrices. The workflow incorporated structural preparation using CHARMM-GUI, implicit-solvent minimization using OpenMM, parallel evaluation of DREIDING and COMPASS II for the protein systems, and reconstruction of the crystalline phase of ID from experimental crystallographic data.

The simulated density of crystalline ID differed by approximately 3.2% from the value calculated from experimental crystallographic data, while the normalized energy values obtained under the NVT and NPT ensembles showed good agreement. These results support the suitability of the reconstructed ID crystal model for the exploratory computational analyses performed in this study.

The thermodynamic descriptors revealed important limitations in the quantitative treatment of the protein-based encapsulating matrix. In particular, the CED and δ values calculated for the protein systems showed pronounced force-field dependence, while the absolute χ values obtained with the Blends model were substantially higher than those reported for comparable drug–excipient systems. Consequently, these parameters should not be interpreted as rigorous quantitative predictions of miscibility. Nevertheless, within the same computational framework, CFP–ID consistently exhibited lower χ and E_mix_ values than MD–ID, indicating comparatively less unfavorable calculated interactions. This relative trend identifies CFP as the candidate warranting further investigation among the two encapsulating materials evaluated.

The complementary docking analysis of free ID with COX-1 and COX-2 showed a relative computational trend qualitatively consistent with published enzyme-inhibition data. However, this analysis was independent of the ID–encapsulant evaluation and should not be interpreted as evidence of drug–matrix compatibility, formulation performance, or preservation of pharmacological activity following microencapsulation.

The principal limitations of the present study include the force-field sensitivity of the protein cohesive properties, the large absolute χ values obtained with the Blends model, the size mismatch between the modeled components, the representation of MD using a short oligomeric chain, and the limited conformational sampling provided by the 100 ps simulations. Accordingly, the present workflow should be regarded as an exploratory computational pre-screening approach, and the relative interaction trends identified here require subsequent experimental validation using actual microencapsulated formulations.

## Figures and Tables

**Table 1:** Energy minimization of indomethacin and maltodextrin.

System	Initial Energy (kcal/mol)	Final Energy (kcal/mol)
Indomethacin	198.189	1.979
Maltodextrin	180,342.719	332.815

**Table 2:** Energy minimization of the crystalline structure of indomethacin (α-polymorph).

System	Initial Energy (kcal/mol)	Final Energy (kcal/mol)	Cell Change
Unit cell (Z = 6)	1437.161	38.822	<2%
Supercell (5 × 2 × 2; 120 molecules)	775.873	772.043	<1%

**Table 3:** Energy minimization of protein structures.

System	Force Field	Initial Energy (kcal/mol)	Final Energy (kcal/mol)
COX-1	DREIDING	48,018.48	3484.02
	COMPASS II	2,621,351.26	−12,967.22
COX-2	DREIDING	46,691.94	3638.09
	COMPASS II	2,699,134.87	−14,103.20
CFP	DREIDING	54,451.45	1721.19
	COMPASS II	2,929,294.28	−14,721.297

COX-1: Cyclooxygenase-1; COX-2: Cyclooxygenase-2; CFP: Cottonseed flour protein.

**Table 4:** Thermodynamic behavior of the systems during the NVT ensemble.

System	Force Field	Average Temperature (K)	Temperature Standard Deviation (K)	Average Potential Energy (kcal/mol)	Average Total Energy (kcal/mol)	Average Simulation-Cell Density (g/cm^3^)
COX-1	DREIDING	303.15	15.799	10,998.417	19,685.965	0.228
	COMPASS II	303.15	14.746	−5814.106	2873.417	0.053
COX-2	DREIDING	303.15	17.323	11,188.402	19,906.616	0.052
	COMPASS II	303.15	14.641	−7054.693	1663.513	0.045
CFP	DREIDING	303.15	15.961	8516.262	17,164.041	0.272
	COMPASS II	303.15	14.291	−7621.617	1026.175	0.065
MD	COMPASS II	302.13	22.011	478.908	688.538	1.000
ID	COMPASS II	303.15	15.706	44.329	81.370	1.458

COX-1: cyclooxygenase-1; COX-2: cyclooxygenase-2; CFP: cottonseed flour protein; MD: maltodextrin; ID: indomethacin. For the protein systems, density refers to the total mass relative to the simulation-cell volume and should not be interpreted as the intrinsic density of the protein.

**Table 5:** Thermodynamic behavior of the systems during the NPT ensemble.

System	Force Field	Average Temperature (K)	Temperature Standard Deviation (K)	Average Potential Energy (kcal/mol)	Average Total Energy (kcal/mol)	Average Simulation-Cell Density (g/cm^3^)
COX-1	DREIDING	303.15	16.634	9883.129	18,570.739	0.680
	COMPASS II	303.15	14.675	−5820.226	2867.286	0.050
COX-2	DREIDING	303.15	17.751	10,839.035	19,557.187	0.107
	COMPASS II	303.15	15.025	−7031.460	1686.779	0.043
CFP	DREIDING	303.15	16.193	6329.937	14,977.730	0.874
	COMPASS II	303.15	14.421	−7584.528	1063.318	0.070
MD	COMPASS II	302.84	24.049	436.776	646.202	1.385
ID	COMPASS II	303.10	17.277	45.021	82.057	1.380

COX-1: cyclooxygenase-1; COX-2: cyclooxygenase-2; CFP: cottonseed flour protein; MD: maltodextrin; ID: indomethacin. For the protein systems, density refers to the total mass relative to the simulation-cell volume and should not be interpreted as the intrinsic density of the protein.

**Table 6:** Cohesive energy density of the evaluated systems.

System	Force Field	CED (J/m^3^)
COX-1	DREIDING	7.547 × 10^7^
	COMPASS II	2.835 × 10^6^
COX-2	DREIDING	7.920 × 10^6^
	COMPASS II	1.648 × 10^6^
CFP	DREIDING	3.162 × 10^8^
	COMPASS II	1.458 × 10^7^
MD	COMPASS II	2.510 × 10^8^
ID	COMPASS II	7.409 × 10^8^

COX-1: cyclooxygenase-1; COX-2: cyclooxygenase-2; CFP: cottonseed flour protein; MD: maltodextrin; ID: indomethacin.

**Table 7:** Hildebrand solubility parameter (δ) of the evaluated structures.

System	Force Field	δ Total (J/cm^3^)^0.5^
COX-1	DREIDING	8.688
	COMPASS II	1.684
COX-2	DREIDING	2.814
	COMPASS II	1.284
CFP	DREIDING	17.783
	COMPASS II	3.819
MD	COMPASS II	15.814
ID	COMPASS II	27.219

COX-1: cyclooxygenase-1; COX-2: cyclooxygenase-2; CFP: cottonseed flour protein; MD: maltodextrin; ID: indomethacin.

**Table 8:** Mixing energy and Flory–Huggins interaction parameter of the indomethacin–encapsulant binary systems.

System (Base–Screen)	E_mix_ (kcal/mol)	χ
CFP–ID	20.795	35.115
MD–ID	24.421	41.239

CFP: cottonseed flour protein; MD: maltodextrin; ID: indomethacin.

**Table 9:** Molecular docking results for indomethacin with COX-1 and COX-2 using the HDOCK server.

	Ranking	1	2	3	4	5	6	7	8	9	10
System											
COX-1-ID	Docking score	−152.45	−140.62	−136.41	−133.16	−132.68	−129.74	−127.79	−127.42	−124.59	−122.64
	Confidence score	0.7317	0.6474	0.6178	0.5951	0.5918	0.5715	0.5581	0.5557	0.5365	0.5235
COX-2-ID	Docking score	−121.24	−112.36	−106.66	−104.63	−102.97	−101.49	−99.95	−96.95	−95.84	−93.89
	Confidence score	0.5142	0.4574	0.4227	0.4107	0.4011	0.3925	0.3832	0.3672	0.3612	0.3508

COX-1, cyclooxygenase-1; COX-2, cyclooxygenase-2; ID, indomethacin.

## Data Availability

The data supporting the findings of this study are presented in the article and its Supplementary Materials. Additional information may be obtained from the corresponding authors upon reasonable request, subject to the availability of the original computational files.

## Supplementary Materials

The following supporting information can be downloaded at: https://www.mdpi.com/article/10.3390/molecules31203674/s1.

## Abbreviations

The following abbreviations are used in this manuscript:

NSAIDs	Nonsteroidal anti-inflammatory drugs
COX-1	Cyclooxygenase-1
COX-2	Cyclooxygenase-2
GI	Gastrointestinal
MD	Maltodextrin
CFP	Cottonseed flour protein
MDS	Molecular dynamics simulations
CED	Cohesive energy density
ID	Indomethacin
NPT	Number of particles, Pressure, Temperature
NVT	Number of particles, Volume, Temperature
