# Supplementary material for: Exploratory In Silico Evaluation of Microencapsulation Matrices for Indomethacin
Source: Molecules. 2026 Oct 14;31(20):3674. doi: 10.3390/molecules31203674 (PMC13707734; doi:10.3390/molecules31203674)
Supplement: Supplementary file 1 [file molecules-31-03674-s001.zip › molecules-4603624-supplementary.pdf]

Supplementary Material - File S1

S1. NVT Equilibration Convergence Profiles

This annex provides the potential energy convergence profiles and dynamic summary statistics obtained during the NVT molecular dynamics equilibration of the protein systems (COX-1, COX-2, and CFP) evaluated with the DREIDING and COMPASS II force fields. For each system, the energy profile as a function of simulation time (Figures S1-S6) and the corresponding thermodynamic summary (Tables S1-S6) are reported. The profiles show an initial relaxation of the potential energy during the early portion of the NVT trajectory, followed by a comparatively stable energy region maintained throughout the remainder of the 100 ps simulation. These profiles provide direct evidence that the potential energy had stabilized within the time scale evaluated before the energetic descriptors used in the subsequent analyses were obtained.

S1.1 Cyclooxygenase-1 (COX-1)

S1.1.1 Force field: DREIDING

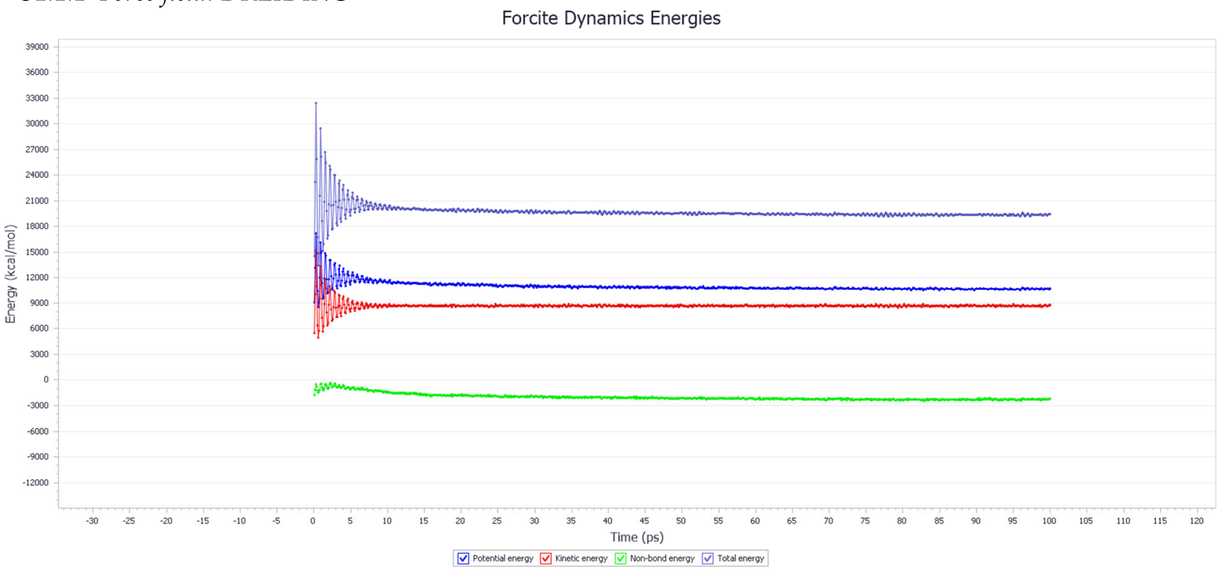

Figure S1. Potential energy convergence profile during NVT equilibration of COX-1 using DREIDING force field.

Total CPU time used by Forcite: 15:36:42 hours.

Table S1. Dynamic Summary of NVT equilibration for COX-1 with DREIDING force field.

	Initial	Final	Average	Standard deviation
Total energy	12,171.534	19,427.433	19,685.965	943.123
Potential energy	3,484.022	10,739.599	10,998.417	564.079
Kinetic Energy	8,687.512	8,687.512	8,687.547	452.747
Total Enthalpy	18,019.082	18,279.156	18,824.734	1,476.022
Temperature	303.150	303.161	303.151	15.799
Pressure	0.081	-0.016	-0.012	0.014
Volume	499,255.186	499,255.186	499,255.186	0.000
Density	0.228	0.228	0.228	0.000

All energies are in kcal/mol, temperature in K, the pressure in GPa, volume in Å^3 and density in g/cm^3.

S1.1.2 Force field: COMPASS II

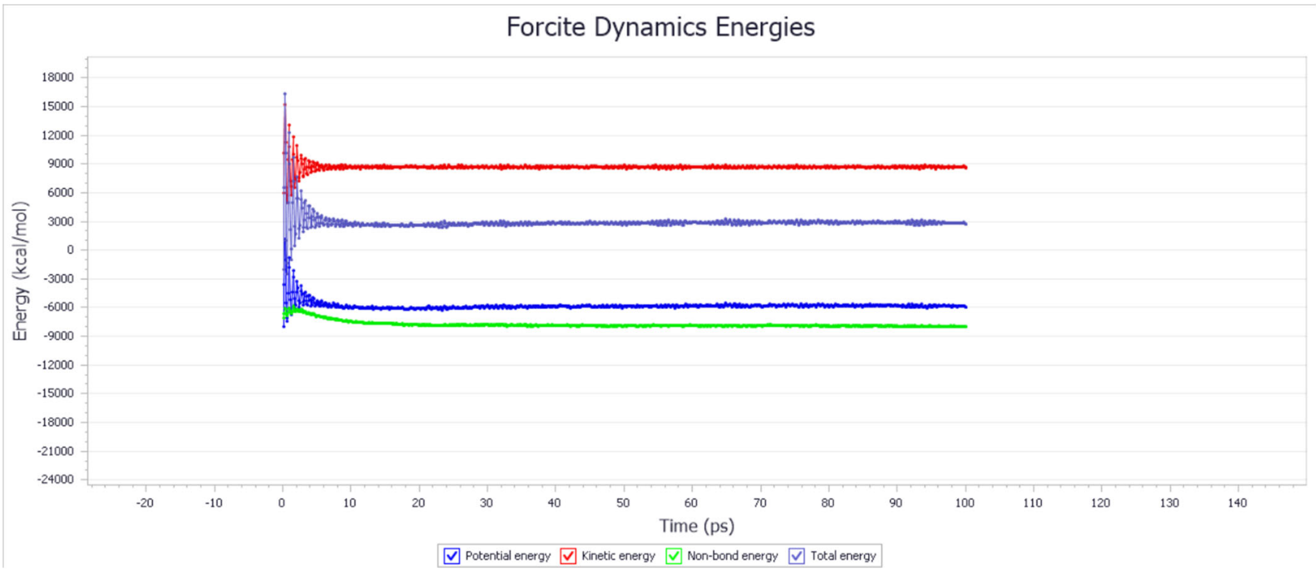

Figure S2. Potential energy convergence profile during NVT equilibration of COX-1 using COMPASS II force field.

Total CPU time used by Forcite: 26:56:50 hours.

Table S2. Dynamic Summary of NVT equilibration for COX-1 with COMPASS II force field.

	Initial	Final	Average	Standard deviation
Total energy	-4,279.704	2,703.622	2,873.417	832.021
Potential energy	-12,967.216	-5,974.989	-5,814.106	462.153
Kinetic Energy	8,687.512	8,678.611	8,687.523	422.597
Total Enthalpy	1,553.087	3,483.840	2,943.363	1,326.922
Temperature	303.150	302.839	303.150	14.746
Pressure	0.019	0.003	0.000	0.003
Volume	2,164,598.555	2,164,598.555	2,164,598.555	0.000
Density	0.053	0.053	0.053	0.000

All energies are in kcal/mol, temperature in K, the pressure in GPa, volume in $Å^3$ and density in g/cm^3.

S1.2 Cyclooxygenase-2 (COX-2)

S1.2.1 Force field: DREIDING

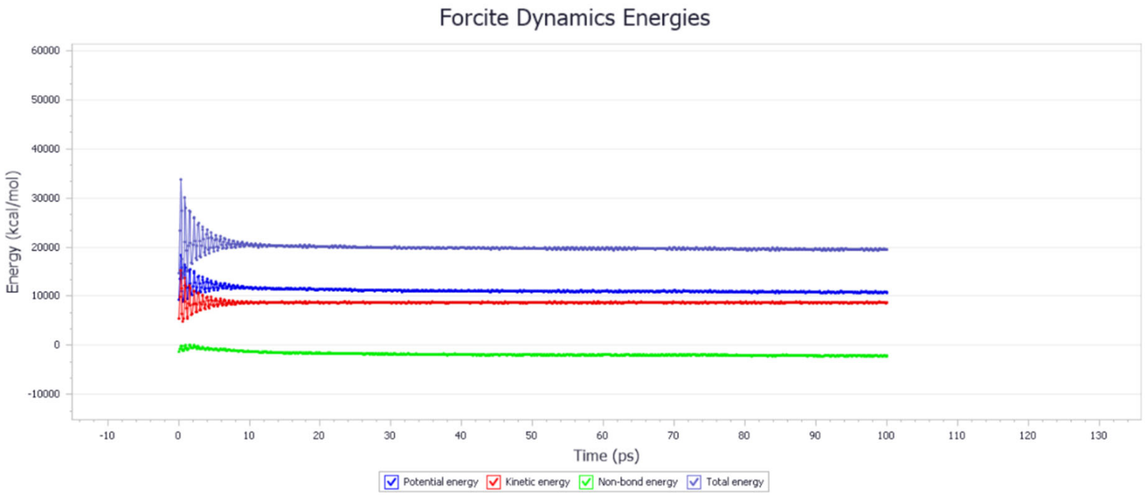

Figure S3. Potential energy convergence profile during NVT equilibration of COX-2 using DREIDING force field.

Total CPU time used by Forcite: 28:11:36 hours.

Table S3. Dynamic Summary of NVT equilibration for COX-2 with DREIDING force field.

	Initial	Final	Average	Standard deviation
Total energy	12,356.333	19,453.026	19,906.616	1,040.643
Potential energy	3,638.097	10,736.710	11,188.402	616.162
Kinetic Energy	8,718.236	8,716.317	8,718.214	498.190
Total Enthalpy	18,165.641	18,550.854	19,143.449	1,564.531
Temperature	303.150	303.083	303.149	17.323
Pressure	0.018	-0.003	-0.002	0.003
Volume	2,210,782.776	2,210,782.776	2,210,782.776	0.000
Density	0.052	0.052	0.052	0.000

All energies are in kcal/mol, temperature in K, the pressure in GPa, volume in $Å^3$ and density in g/cm^3.

S1.2.2 Force field: COMPASS II

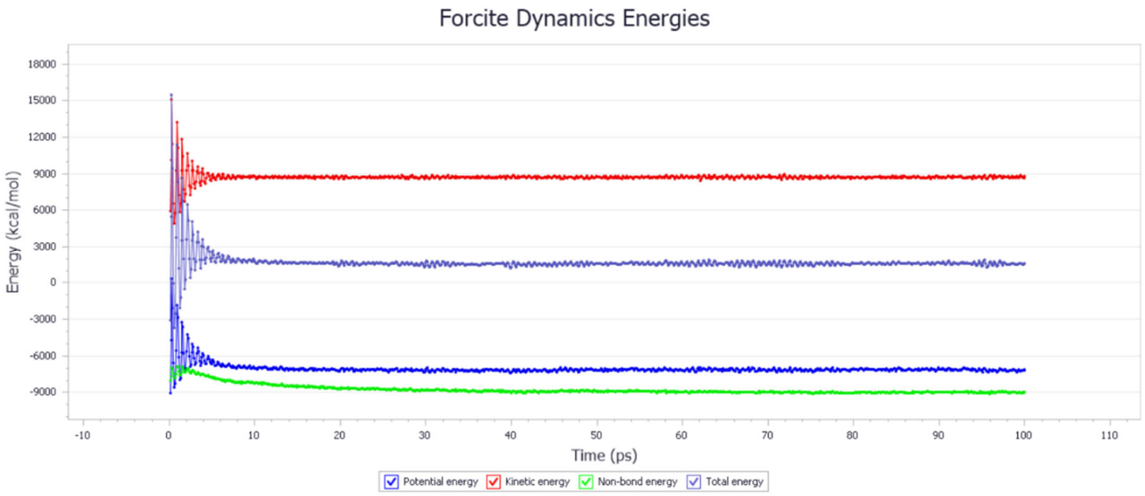

Figure S4. Potential energy convergence profile during NVT equilibration of COX-2 using COMPASS II force field.

Total CPU time used by Forcite: 30:10:38 hours.

Table S4. Dynamic Summary of NVT equilibration for COX-2 with COMPASS II force field.

	Initial	Final	Average	Standard deviation
Total energy	-5,384.967	1,593.995	1,663.513	853.265
Potential energy	-14,103.203	-7,087.745	-7,054.693	493.682
Kinetic Energy	8,718.236	8,681.740	8,718.206	421.053
Total Enthalpy	425.066	2,364.334	1,762.553	1,359.131
Temperature	303.150	301.881	303.149	14.641
Pressure	0.016	0.002	0.000	0.003
Volume	2,550,883.538	2,550,883.538	2,550,883.538	0.000
Density	0.045	0.045	0.045	0.000

All energies are in kcal/mol, temperature in K, the pressure in GPa, volume in $Å^3$ and density in g/cm^3.

S1.3 Cottonseed Flour Protein (CFP)

S1.3.1 Force field: DREIDING

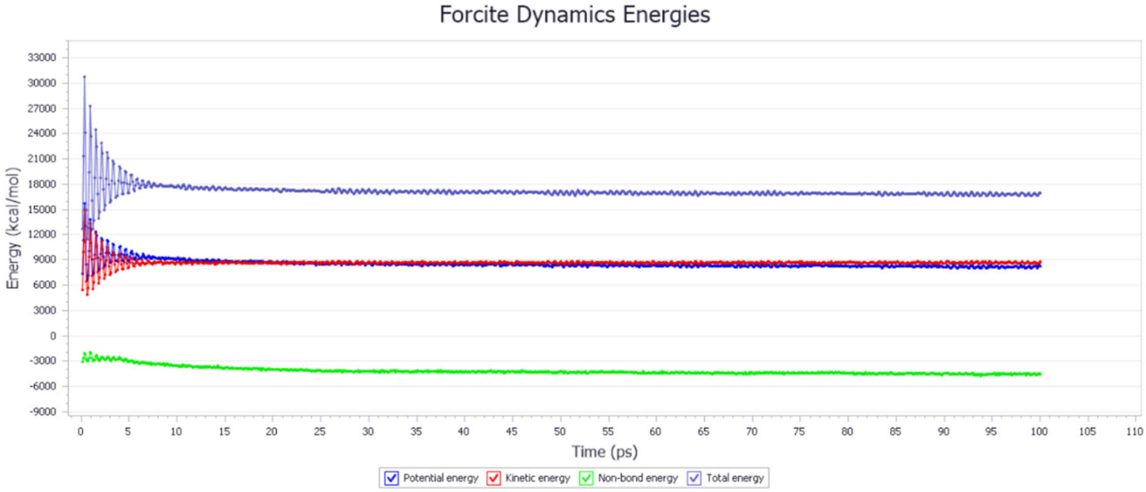

Figure S5. Potential energy convergence profile during NVT equilibration of CFP using DREIDING force field.

Total CPU time used by Forcite: 12:19:58 hours.

Table S5. Dynamic Summary of NVT equilibration for CFP with DREIDING force field.

	Initial	Final	Average	Standard deviation
Total energy	10,368.948	16,977.644	17,164.041	949.864
Potential energy	1,721.196	8,217.355	8,516.262	576.773
Kinetic Energy	8,647.753	8,760.289	8,647.779	455.309
Total Enthalpy	16,137.080	17,530.109	15,388.866	1,469.342
Temperature	303.150	307.095	303.151	15.961
Pressure	0.094	0.009	-0.029	0.016
Volume	426,320.857	426,320.857	426,320.857	0.000
Density	0.272	0.272	0.272	0.000

All energies are in kcal/mol, temperature in K, the pressure in GPa, volume in Å^3 and density in g/cm^3.

S1.3.2 Force field: COMPASS II

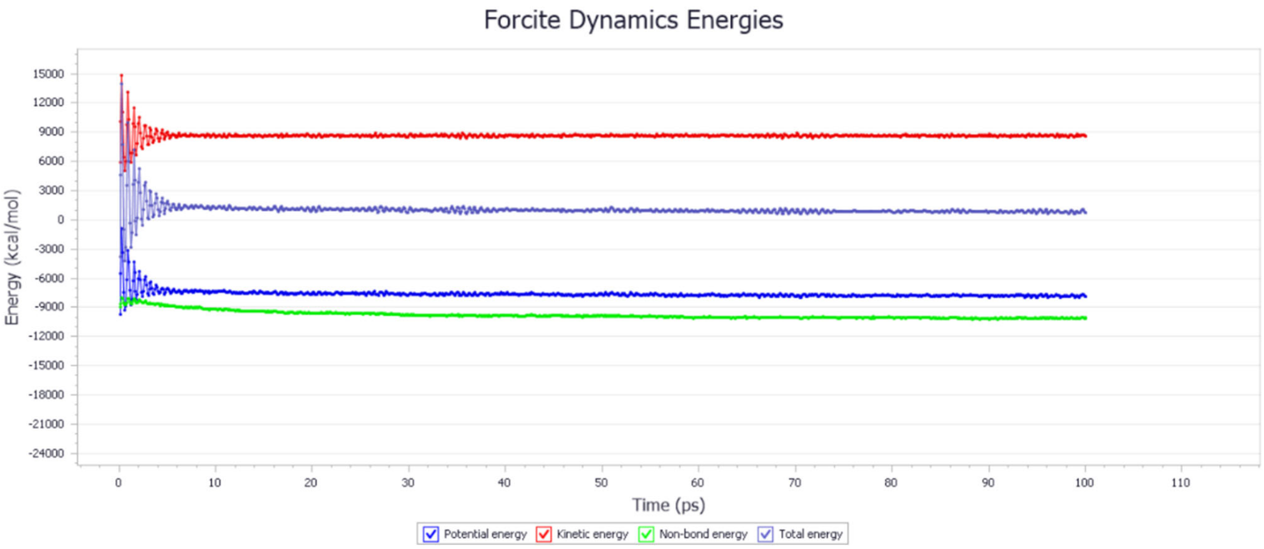

Figure S6. Potential energy convergence profile during NVT equilibration of CFP using COMPASS II force field.

Total CPU time used by Forcite: 17:54:13 hours.

Table S6. Dynamic Summary of NVT equilibration for CFP with COMPASS II force field.

	Initial	Final	Average	Standard deviation
Total energy	-6073.543	717.520	1,026.175	786.237
Potential energy	-14,721.295	-7835.154	-7621.617	428.275
Kinetic Energy	8,647.753	8,552.673	8,647.791	407.663
Total Enthalpy	-334.564	465.745	936.125	1,264.416
Temperature	303.150	299.817	303.151	14.291
Pressure	0.022	-0.001	0.000	0.004
Volume	1,779,365.115	1,779,365.115	1,779,365.115	0.000
Density	0.065	0.065	0.065	0.000

All energies are in kcal/mol, temperature in K, the pressure in GPa, volume in $Å^3$ and density in g/cm^3.

NOTE: Structural RMSD profiles could not be generated because the trajectory files required for this analysis were not retained for the protein NVT simulations. Therefore, structural convergence could not be independently assessed by RMSD. The potential-energy profiles presented in Figures 1–6 nevertheless show an initial relaxation followed by a comparatively stable energy region over the remainder of the 100 ps NVT simulations. Accordingly, these profiles support energetic stabilization within the simulated time scale, while the absence of RMSD analysis and the limited 100 ps trajectory length are acknowledged as limitations of the present exploratory computational study.
